# Unchain My Blood: Lessons Learned from Self-Assembled Dendrimers as Nanoscale Heparin Binders

**DOI:** 10.3390/biom9080385

**Published:** 2019-08-20

**Authors:** Domenico Marson, Erik Laurini, Suzana Aulic, Maurizio Fermeglia, Sabrina Pricl

**Affiliations:** Molecular Biology and Nanotechnology Laboratory (MolBNL@UniTS), Department of Engineering and Architecture, University of Trieste, 34127 Trieste, Italy

**Keywords:** dendrimers, amphiphilic dendrons, self-assembled dendrimers, heparin, anticoagulant activity reversal, protamine replacers, molecular simulations

## Abstract

This review work reports a collection of coupled experimental/computational results taken from our own experience in the field of self-assembled dendrimers for heparin binding. These studies present and discuss both the potentiality played by this hybrid methodology to the design, synthesis, and development of possible protamine replacers for heparin anticoagulant activity reversal in biomedical applications, and the obstacles this field has still to overcome before these molecules can be translated into nanomedicines available in clinical settings.

## 1. Introduction

**Figure 1:**
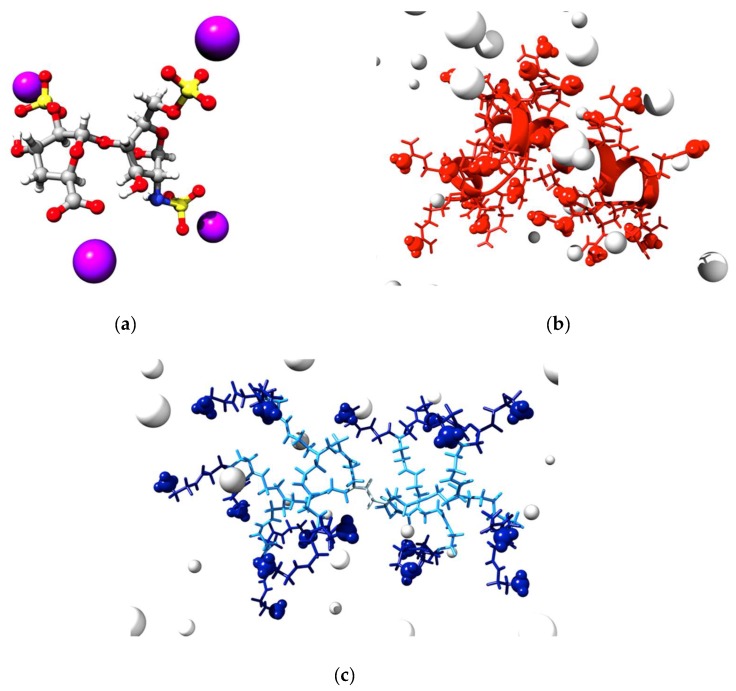
(**a**) Molecular model of the average repeating unit of heparin shown as atom-colored sticks-and-balls (**c**), gray; O, red; N, blue; S, yellow; H, white. Na^+^ counterions are portrayed as purple spheres. (**b**) Molecular model of protamine structure in physiological solution. The small protein backbone is evidenced by a red ribbon, while the residues are shown as red sticks. One of the guanidinio –NH_2_ groups is highlighted using red spheres. (**c**) Molecular model of a generation 2 (G_2_) ethylenediamine (EDA)-core poly(amidoamine) (PAMAM) dendrimer structure in physiological solution. The dendrimer EDA core is shown as light blue sticks, the branching units are depicted as dodger blue sticks, while the branches and the relevant surface amine groups, positively charged at pH 7.4, are highlighted as navy blue spheres. In panels **b**,**c**, Na^+^ and Cl^−^ ions and counterions are represented by small and large white spheres, respectively, while water molecules are not shown for clarity.

In healthy individuals, blood freely circulates along arteries and veins throughout the entire human body, in which the normal vascular endothelium acts as an overall antithrombotic surface. Yet, it becomes immediately active if the hemostatic system is triggered by the coagulation cascade. As we know, when a blood vessel is damaged platelets and fibrin quickly engage in an aggregation process leading to hemorrhage prevention. This is beneficial in avoiding blood loss, at the same time excessive clotting can lead to life-threatening thrombotic complications. Such a physiological situation might disgracefully happen during different clinical practices, such as large surgeries, blood transfusions, or dialysis treatments; accordingly, a delicate balance between thromboembolism risk and excessive bleeding prevention must be carefully optimized for each patient. Heparin, a linear polysaccharide consisting of repeating units of 2-*O*-sulfated iduronic acid and 6-*O*-sulfated, *N*-sulfated glucosamine (IdoA(2S)-GlcNS(6S) (Figure 1a), is one of the most charged dense naturally occurring polyanion in biological systems [1] and, equally, one of the most widely used clinical anticoagulants worldwide [2]. Once the clinical treatment requiring coagulation control is over, reversal of the administered heparin anticoagulant activity is obviously required. Protamine, a small, nuclear, basic, arginine-rich protein (Figure 1b), is the only FDA (Food and Drug Administration) approved molecule employed to that purpose. In fact, by virtue of its high positive charge, protamine binds to heparin via strong electrostatic interaction thereby removing the polysaccharide from the bloodstream and ultimately re-enabling clotting. Unfortunately, protamine is associated with several, important side-effects, including immunological and inflammatory alterations, and anaphylactic responses characterized by hypotension, bradycardia, pulmonary vasoconstriction, and allergy if not exactly administered [3]. Hence, the development of new, protamine-alternative heparin-rescue agents, which could stably bind the polyanion so that excretion of their intact complexes can easily and quantitatively occur, but at the same time, could safely degrade into non-toxic components if administered in excess, is a current hot need in medical practice.

DNA is indisputably another archetypal charge-dense polyanion that dominates biology. Being the guardian of the genetic information in humans and almost all other organisms, it is likely the most studied biological macromolecule. And, since upon unlocking the secrets of the human genome it was discovered that nearly all diseases have a genetic component, the biomedical interest in DNA for gene therapy has dramatically increased in recent years [4]. The Food and Drug Administration (FDA) defines gene-based therapeutics as “products that mediate their effects by transcription and/or translation of transferred genetic material and/or by integrating into the host genome and that are administered as nucleic acids, viruses, or genetically engineered micro-organisms. The products may be used to modify cells in vivo or transferred to cells ex vivo prior to administration to the recipient [5]”. Thus, the opportunity to treat such disorders by replacing the defective gene(s) with normal (healthy) one(s) offers a novel therapeutic approach for patients who suffer from such diseases. However, to be successful in the clinics gene, therapy largely relies on the adoption of safe, effective, and efficient viral/non-viral nanovectors to transfer DNA to mammalian cells both ex vivo and in vivo [6]. In the plethora of currently available nanocarriers for gene delivery dendrimers, i.e., nanosized, hyperbranched, and self-similar molecules represent prototypical vectors with fine-tunable properties for optimal DNA delivery [7]. Their peculiar structure is constituted by three distinct domains (Figure 1c): (1) A fundamental atom, or most frequently, a group of atoms defined as the core; (2) the branching units, which, emanating from the core through diverse chemical reactions, allow the dendrimeric molecule to grow in geometrically organized radial layers known as generations (G); and (3) an exponentially increasing number peripheral surface groups, which constitute a multivalent nanoscale array, and can therefore form high-affinity interactions with a variety of biological targets, including nucleic acids [8,9,10]. However, high generation dendrimer synthesis is extremely laborious and time-consuming, since the final product purification is difficult and hampered by the presence of highly similar side products. Thus, notwithstanding the highly promising results achieved with these molecules, the difficulties inherent in large-scale good manufacturing practice (GMP) production of high generation dendrimers are hampering their way to the clinics [11]. One way to circumvent these obstacles and to make multivalent systems which are synthetically simpler and more responsive is to design low molecular weight amphiphilic dendrons which, upon spontaneous self-assembly into dendrimer-like nanovectors, can mimic (and eventually outperform) their covalent counterparts in size, shape, and functions [12,13,14,15,16,17,18,19,20].

In the panorama described above, during the last 5 years our laboratory, in collaboration with different international groups, has designed and produced a series of amphiphilic molecules bearing dendritic portions as polar heads and various hydrocarbon chains as hydrophobic moieties, able to self-organize into supramolecular nanostructures of different size and shape with the unique capability of selectively binding the two major polyanions, heparin and DNA [21,22,23,24,25,26,27,28,29]. This, with the goal of employing the resulting self-assembled dendrimers as protamine replacer and gene delivery nanovectors, respectively. This short review summarizes our efforts focused on the first, less popular but certainly not less important field in the hope of paving the way for new heparin antidots to reach the stage of clinical nanomedicine.

## 2. Can Covalent Poly(Amidoamine) Dendrimers Efficiently Bind Heparin as Protamine Replacers?

As mentioned in the previous section, protamine is the only clinically approved agent to be employed in heparin anticoagulant activity reversal during chirurgical and medical practices. Derived from shellfish, this small arginine-rich cationic protein causes adverse reactions in nearly 10% of patients, and up to 2.6% of cardiac surgeries experience serious complications due to protamine suboptimal administration [30]. Since covalent dendrimers can mimic many aspects of protein behavior [31], we reasoned that these hyperbranched molecules, and particularly the poly(amidoamine) (aka PAMAM) dendrimers originally reported by Don Tomalia in 1985 [32], which feature positively charged groups on their outer surface, could work well as heparin ligands. Thus, we began our journey in the quest of possible protamine replacers by studying heparin binding by ethylenediamine (EDA)-core PAMAMs with different branching generations (G_0_–G_6_) by means of a combined experimental/computational approach [33].

**Figure 2:**
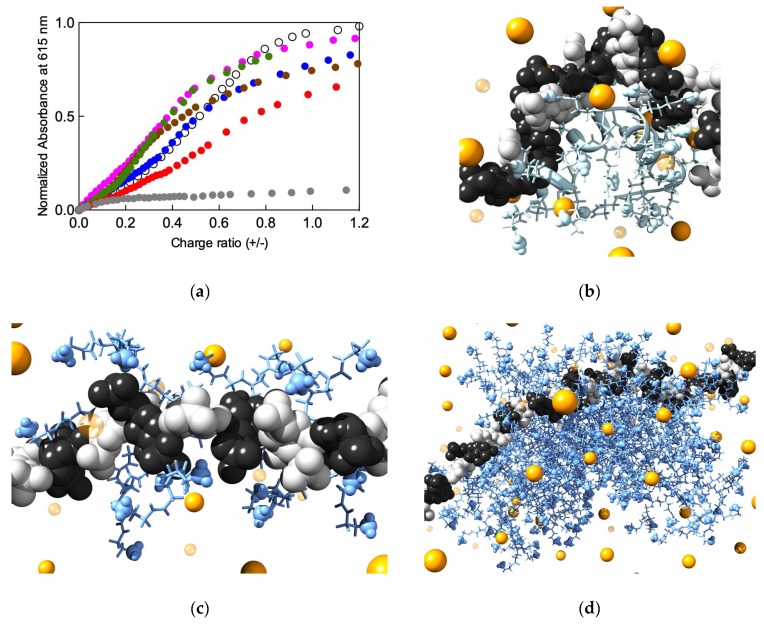
(**a**) Heparin binding curves of protamine (black empty symbols) and EDA-core PAMAM dendrimers from G_0_ to G_6_ (colored filled symbols) obtained from the Mallard Blue (MB) dye displacement assay performed in buffer (10 mM Tris HCl, pH 7.4) containing 150 mM NaCl. Color legend: Gray, G_0_; red, G_1_, green, G_2_, brown, G_3_, blue, G_4_, and pink, G_6_). The binder concentration on the x-axis is expressed in terms of binder/heparin charge ratio, in which the average negative charge of the heparin disaccharide unit is taken equal to the ideal value of −4. The different binder charges are listed in the second column of Table A1. Equilibrated MD (Molecular Dynamics) simulation images of (**b**) protamine, (**c**) G_2_, and (**d**) G_6_ EDA-core PAMAM dendrimers in complex with heparin. In all panels, heparin is shown as white (D-glucosamine) and black (L-iduronic acid) spheres, while protamine and dendrimers are portrayed as light blue sticks, with the positively-charged groups highlighted as spheres. Some Na^+^ and Cl^−^ used as counterions and to mimic the 150 mM ionic strength in solution are shown as small and large yellow spheres, respectively. Water molecules are omitted for clarity. Adapted from [33] with the permission of The Royal Society of Chemistry.

The investigation started with an experimental heparin binding competition assay (HBCA) exploiting Mallard Blue (MB), a deep-blue colored, positively charged (+5) synthetic dye based on an arginine-functionalized thionine. This compound was specifically developed by our group for ultra-high-affinity sensing of clinically-relevant heparin levels in both physiological solutions and serum [34]. The dye reports on heparin levels by quantitatively binding to the polyanion; this, in turn, results in a significant change in its UV−visible spectroscopic profile. Based on this evidence, we conceived the HBCA as follows: First, two solutions containing heparin and MB are mixed and then the resulting system is gradually titrated with solutions containing protamine and PAMAM dendrimers of varying generations. In the presence of increasing concentration of each possible heparin binder, the dye is gradually displaced from heparin and the absorbance intensity of free MB increases accordingly, as shown in Figure 2a. Curve fitting of the data shown in this figure allowed us to calculate the main binding parameters, i.e., the effective heparin binder concentration require to displace 50% of the MB dye from the polyanion (EC_50_), and the charge excess, that is the number of binder positive charges per heparin negative charge (CE_50_) required to attain 50% displacement of the MB dye. These values are listed in Table A1.

From Figure 2a and Table A1 we see that the smallest dendrimer of the series (G_0_) is clearly not able to displace MB from the polysaccharide. On the other hand, as the dendrimer generation grows, the corresponding EC_50_ value decreases (from 10.10 µM of G_1_ to 0.22 µM of G_6_) by virtue of the increasing dendrimer surface charge leading to higher heparin/binder affinity. However, in terms of a given dendrimer’s ability to exploit each individual positive charge in heparin binding (CE_50_), the observed behavior is not monotonic, in that both the smallest (G_1_) and the biggest (G_6_) dendrimers are characterized by the worst (i.e., highest) EC_50_ value, while the other PAMAMs (G_2_–G_4_) exhibit better heparin binding performance in this respect (Table A1). An utterly analogous trend is observed when considering the ligand dose required to bind 100 IU of heparin (last column in Table A1). The touchstone molecule, protamine, is characterized by an EC_50_ value of 2.34 µM, a CE_50_ of 0.52, and a corresponding dose of 0.32 mg/100 heparin IU (Table A1). Therefore, a global reconsideration of all values listed in Table A1 leads to the conclusion that the most promising protamine replacer among the entire PAMAM dendrimer series considered is G_2_, not only because its heparin binding parameter set (EC_50_ = 2.55 µM and CE_50_ = 0.38) and dosage required (0.25 mg/100 heparin IU) most favorably compare with those characterizing the small arginine-based protein but also because low generation dendrimers are generally endowed with lower in vivo toxicity effects [35].

This experimental evidence was rationalized using computer-based molecular dynamics (MD) simulations (see Figure 2b–d). To the purpose, we exploited the concept of effective free energy of binding ∆G_bind,eff_, that is the specific energetic contribution to each heparin/binder complex formation afforded only by those dendrimer (or protamine) charges in constant and productive interaction with the polyanion. The analysis of each heparin/binder nanoassembly MD simulation allowed us to precisely identify and quantify these dendrimer (protamine) positively-charged groups (N_eff_), estimate their individual contribution to the overall binding energy, and cumulatively express this as ∆G_bind,eff_, as shown in Table A2. Yet, to be able to compare simulation data both among themselves and with experimental CE_50_ values, we further introduced the concept of effective-charge-normalized binding free energy, i.e., ∆G_bind,eff_/N_eff_, as shown in the last column of Table A2. As a result of these in silico experiments, it appears evident that although 6 out of 8 positive charges of the small G_1_ dendrimer are productively engaged in heparin binding, their overall effective binding contribution is quite low, just −0.19 kcal/mol (Table A2). On the other hand, the largest G_6_ dendrimer, despite its plethora of positively-charged amine termini (+256), is able to organize only 45 of them for best productive heparin binding (Figure 2d), most likely because of its intrinsic high molecular rigidity. Accordingly, this big hyperbranched molecule is a sub-optimal polyanion binder (∆G_bind,eff_/N_eff_ = −0.40 kcal/mol, Table A2). The G_3_ and G_4_ PAMAMs behave quite similarly to each other, with 15 and 16 positive charges in contact with heparin, respectively, ultimately resulting in comparable, intermediate effective-charge-normalized affinity values (−1.06 and −0.91 kcal/mol, respectively, Table A2). Finally, the last G_2_ dendrimer (Figure 2c) is not only able to exploit 13/16 terminal groups in complexing heparin but it also does so in the most effective way (∆G_bind,eff_/N_eff_ = −1.30 kcal/mol, Table A2), in agreement with the corresponding experimental CE_50_ value (Table A1). If compared with the predicted values for protamine (N_eff_ = 12 over 24 available charges, and ∆G_bind,eff_/N_eff_ = −0.33 kcal/mol, Table A2), it appears that the in silico analysis also identifies the EDA-core G_2_ PAMAM dendrimer as the preferred polyanion binder in the search of an alternative heparin anticoagulant activity reversal agent.

With this double information at hand, we next verified whether the G_2_ PAMAM could still operate heparin binding in the presence of serum, and compared the relevant results with those obtained using protamine in the same environment. Very pleasingly we found that, under these substantially more challenging conditions, while protamine worsened its performance (EC_50,serum_ = 3.51 µM, CE_50,serum_ = 0.79, and dose = 0.49 mg/100 heparin IU) the G_2_ dendrimer slightly enhanced its binding affinity for the polyanion (EC_50,serum_ = 2.15 µM, CE_50,serum_ = 0.32, and dose = 0.21 mg/100 heparin IU). Notwithstanding these highly encouraging results we ultimately reasoned that, even if low generation PAMAM dendrimers are often overlooked for biomedical applications despite their good toxicological profiles [35], a substantially more degradable, less expensive, and easier-to produce system could be more desirable for safe and effectual production under good-manufacturing-practice (GMP) quantities required for the translation of a new protamine replacer to the clinic. Bearing this belief in mind, we then plunged ourselves into the sea of self-assembly nanoscale multivalent surfaces in the hope of finding an alternative, even more responsive, synthetic, protamine-mimic heparin binder.

## 3. Self-Assembled Dendrimers as Protamine Replacers

### 3.1. **C_22_G_1_**, the First Example of Performing Heparin-Binding Self-Assembled Dendrimer

In the last decade, our group has been quite active in several international projects dealing with the development of amphiphilic dendrons that, upon self-assembling into micellar spherical pseudo-dendrimers, exhibited unique binding properties towards nucleic acids which entailed them to be exploited as nanocarriers for gene delivery in cancer therapeutics [18,19,20,22,23,25,27,28]. Notably, such systems are synthetically straightforward, with programmed self-assembly of simple building blocks being used as the key nanofabrication step. Since DNA and RNA are also polyanions, we alleged that dendrimer-like micelles originating from the self-organization of amphiphilic dendrons could potentially show high-affinity heparin binding and, as such, act as protamine mimetics.

Therefore, drawing from previous experience, we begin our investigation in the field with **C_22_G_1_**, an amphiphilic dendron featuring two polar head groups constructed from *N*,*N*-di(3-aminopropyl)-*N*-methylamine (DAPMA) and an apolar tail constituted by a C_22_-long hydrocarbon chain. These molecules readily self-assemble into micellar structures with a critical micelle concentration (CMC) of 4 µM in phosphate buffer saline at physiological pH [36]. Moreover, preliminary heparin binding assays conducted in pure water confirmed the affinity of **C_22_G_1_** micelles for this anionic polysaccharide. Accordingly, we embarked in a thorough study aimed at fully characterizing these nanostructures in bio-relevant media, investigating their heparin-binding properties in such challenging conditions, and, above all, determining their functional heparin reversal in comparison with the gold-standard protamine and our covalent G_2_ PAMAM dendrimer alternative.

#### 3.1.1. **C_22_G_1_** Self-Assembled Dendrimers Have High Heparin Affinity at Physiological Ionic Strength

**Figure 3:**
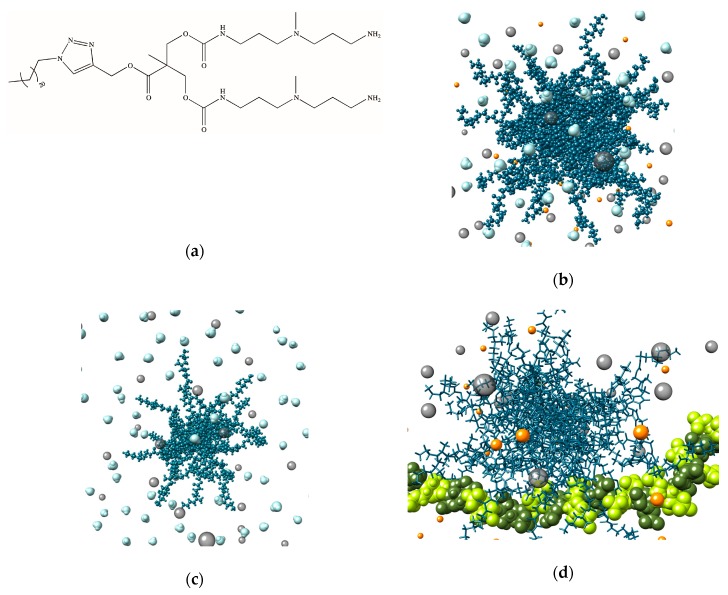
(**a**) Chemical structure of the amphiphilic dendron **C_22_G_1_**. Atomistic models of the nanomicelles formed by **C_22_G_1_** under 150 mM NaCl (**b**) and no-salt (**c**) conditions, as obtained from multiscale molecular simulations. In panels (**b**) and (**c**) the **C_22_G_1_** molecules appear as steel blue sticks-and-balls while some representative water molecules, Na^+^, and Cl^−^ ions are also shown as light blue, orange, and gray spheres, respectively. (**d**) Atomistic model of a **C_22_G_1_** self-assembled dendrimer in complex with heparin under 150 nM NaCl conditions. In this panel, the **C_22_G_1_** molecules are portrayed as steel blue sticks, while heparin is shown as dark olive green (l-iduronic acid) and light green (d-glucosamine) spheres. Water molecules are omitted for clarity while some Na^+^ and Cl^−^ ions are shown as orange and gray spheres, respectively. Adapted from [21] with the permission of The Royal Society of Chemistry.

Since the assays previously employed to monitor aggregation and heparin binding of the selected **C_22_G_1_** amphiphilic dendron (Figure 3a) were carried out either in pure water or at very low salt concentration (≤5 mM) [36], in the first place we decided to investigate in detail the behavior of this molecule in more biologically relevant media. To the purpose, we initially gained information of the generation of **G_22_G_1_** self-assembled nanostructures under different ionic strengths using computer-assisted multiscale molecular simulations [21]. In particular, exploiting a combination of atomistic and mesoscale modeling we predicted that at the physiological salt concentration (150 mM NaCl), **C_22_G_1_** self-organizes into well-defined spherical micelles characterized by an aggregation number (N_agg_) of 24 ± 1, a total surface charge (N_tot_) of +96 ± 4, and an average micellar diameter (D_m_) of 9.3 ± 0.1 nm (Figure 3b). Contextually, when **C_22_G_1_** was challenged in silico for self-assembling under no-salt conditions, simulations still anticipated the formation of spherical nano-objects, yet with substantially smaller N_agg_ (11 ± 3), N_tot_ (44 ± 12), and D_m_ (6.3 ± 0.5 nm) (Figure 3c). The predicted increase of the micellar aggregates in response to increasing ionic strength was attributed to the salt-mediated screening of the micellar surface charge paralleled by the increasing contribution of the hydrophobic interactions. These two effects, acting in synergy, allow a larger number of individual dendrons to be incorporated into the nanomicelles, ultimately leading to larger nanoassemblies. Interestingly, at variance with the covalent G_2_ PAMAM molecule discussed above, the non-covalent nature of the **G_22_G_1_** nanostructures endows them with the ability to respond to this physiologically-relevant environmental stimulus by modifying their characteristic dimensions to an extent covalent would be unable to reach.

The changes in micellar dimensions envisaged by computer simulations were experimentally confirmed by dynamic light scattering (DLS) measurements performed under the same conditions. Indeed, in the presence of 150 mM NaCl the measured average micellar diameter D_m_ was equal to 9.1 ± 0.1 nm whilst in the absence of salt the D_m_ value reduced to 5.8 nm, in excellent agreement with both theoretical predictions. Given the established increase in the **G_22_G_1_** nanomicelle dimensions under physiological salt conditions, and the further evidence—based on Nile Red solubilization assay—that these amphiphilic dendrons could still self-organize in the presence of heparin with a CMC of 14 µM, we surmised that these properties could be beneficial to its heparin binding ability. Therefore, we evaluated the parameters (EC_50_, CE_50_, and the related dose) of this self-assembled dendrimer for heparin binding in 150 mM NaCl buffered solution at pH 7.4 (again using our MB displacement assay) and compared the results with those obtained both with protamine and the covalent G_2_ PAMAM molecule. As seen in the upper part of Table 1, the EC_50_ values indicate that protamine and the small PAMAM dendrimer apparently are a more effective heparin binder than the C_22_G_1_ nanomicelles, for which EC_50_ is approximately 3 times higher than for the other two compounds.

However, the key parameter to be used for an objective comparison among these three quite dissimilar heparin binders is CE_50_, which is their charge efficiency. In this case, from Table 1 it is quite evident that the **C_22_G_1_** self-assembled dendrimer (CE_50_ = 0.28) substantially outperforms both protamine (0.52) and G_2_ PAMAM (0.38) in polyanion binding affinity. Also, according to this assay, the **C_22_G_1_** nanomicelles are active at a dose (0.23 mg/100 heparin IU) significantly lower than protamine (0.32 mg/100 heparin IU) and even slightly lower than that required by the alternative, covalent molecule (0.25 mg/100 heparin IU).

Molecular simulations were again employed to support the experimental binding evidence, and the relevant results are shown in Table 2.

Table 2 indicates that, under physiological salt concentration, **C_22_G_1_** micelles productively expose only 33% of their global 96 positive charges for effective heparin binding, compared to the 50% and 80% of the charged groups exploited by protamine and the G_2_ PAMAM dendrimer, respectively. Notwithstanding, the highly flexible nature of the **G_22_G_1_** self-assembled structures endows them with the ability to adopt the most effective conformation for heparin binding and, in so doing, to present each individual charge for the most efficient polyanion interaction (Figure 3d). Ultimately, this translated in the most favorable heparin effective-charge-normalized free energy binding (∆G_bind,eff_/N_eff_) value (−2.03 kcal/mol) for the **C_22_G_1_** self-assembled dendrimer along the entire binder series, in agreement with the corresponding experimental data.

#### 3.1.2. Human Serum Is a (Moderate) Achille’s Heel in **C_22_-G_1_** Self-Assembled Dendrimers Heparin Binding

Human serum undoubtedly represents the most biologically relevant and, contextually, the most challenging medium for probing the efficacy of alternative heparin binders. Indeed, with the exception of proteins involved in blood clotting, serum is populated by all other proteins and components (e.g., antibodies, antigens, hormones, and endogenous/exogenous species) which are routinely found in blood. Therefore, the next step in the evaluation of **C_22_G_1_** self-assembled dendrimers as potential protamine replacers in clinical applications consisted in performing MB displacement assays in the presence of 100% human serum. The lower part of Table 1 illustrates the relevant results, again in comparison with those obtained for protamine and G_2_ PAMAM under the same experimental conditions. Rather dismayingly, human serum somewhat abated the heparin binding ability of the **C_22_G_1_** self-assembled dendrimers, resulting in an increase of the relevant CE_50_ value to 0.96 (from 0.28 in buffered salted solution, upper part of Table 1). On the other hand, the CE_50_ for protamine was also negatively affected, albeit to a lesser extent (0.79 in serum vs. 0.52 in salted buffer, Table 1) while G_2_ PAMAM was equally if not even performing more in these challenging conditions (CE_50_ = 0.32, Table 1 and Section 2).

These results clearly highlight a major difference in the behavior of covalent and self-assembled nanostructures: While in human serum the former can withstand interfering blood components during heparin binding, the latter ones loose (at least in part) their polyanion binding ability when tested in competitive media. We ascribed the reason of this suboptimal performance to a partial disaggregation of the **C_22_G_1_** self-assembled dendrimers (most likely operated by human albumin, the highly negatively charged, most abundant serum protein with specific binding sites for hydrophobic units) into their individual building blocks. As such the isolated **C_22_G_1_** molecules, we verified (data not shown), were indeed unable to bind heparin effectively. Nonetheless, since the **C_22_G_1_** self-assembly was not abrogated but only moderately compromised in the presence of serum with respect to the covalent counterparts, we considered the in-serum heparin binding performance of our systems still effective (CE_50_ < 1) and that the use of self-assembled dendrimers for this specific application could ultimately bring further advantages, as discussed below.

#### 3.1.3. **C_22_-G_1_** Nanomicelles Can Be Degraded and Disassembled at Physiological pH but Are Stable in the Presence of Heparin and Can Reverse its Anti-Coagulant Effect

**Figure 4:**
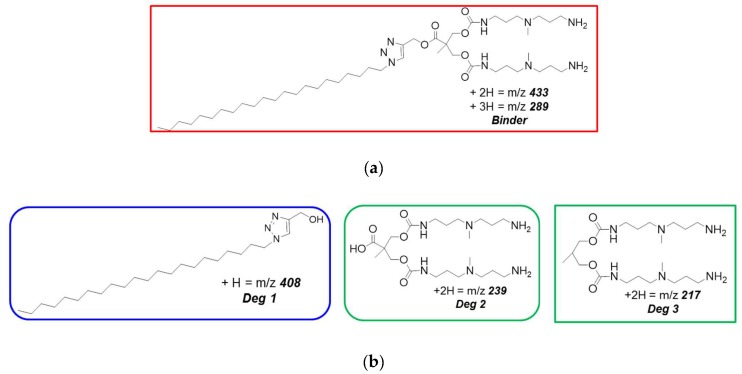
Structure of the molecular ions associated with the initially intact **C_22_G_1_** molecule (**a**) and with the final ester hydrolysis products (**b**) identified in the amphiphilic dendron degradation pathway along the time course of the electrospray mass spectrometry (ESMS)-based assay using a solution of **G_22_G_1_** originally dissolved into ammonium bicarbonate at pH = 7.4. Adapted from [21] with the permission of The Royal Society of Chemistry.

As mentioned in Section 1, once a given medical procedure in which heparin is used for clotting prevention is concluded, there is the immediate need to neutralize the polyanion anti-coagulant effect to allow blood clotting and recovery to begin. This is achieved by treating the patient with the only FDA approved heparin reversal compound, protamine, which requires a rigorous and personalized administration to avoid, or at least, minimize the deleterious side-effects. Therefore, a protamine replacer which, in addition to the required heparin binding characteristics, can safely degrade into non-toxic components if administered in excess could represent an ideal alternative heparin antidote. As it can be noticed from Figure 3a, the **C_22_G_1_** molecular structure features one ester group in its central, linker part, which was incorporated by design with the idea of making this dendritic scaffold degradable via hydrolysis of this moiety in the presence of biological triggers (e.g., pH or esterases). We also surmised that, should our G_22_G_1_ dendron break down over time in a controllable and predictable way into smaller subunits, this could ultimately enhance its biocompatibility, lower its toxicity, and limit its persistence in cells. Moreover, dendron degradation would also disassemble the multivalent heparin binding array, thereby acting as an effective way of “switching off” its biological activity. To verify whether our molecular design and the underlying hypotheses were correct, we conducted an electrospray mass spectrometry (ESMS)-based assay to probe the pathways of dendron degradation which were actually taking place at pH = 7.4 [15,21]. While at the initial stages of the experiment the molecular ions associated with the intact **C_22_G_1_** molecule (Figure 4a) were clearly visible after 24 h they completely disappeared, the dominant peaks in the spectra being those corresponding to the ester hydrolysis products (see Figure 4b).

**Figure 5:**
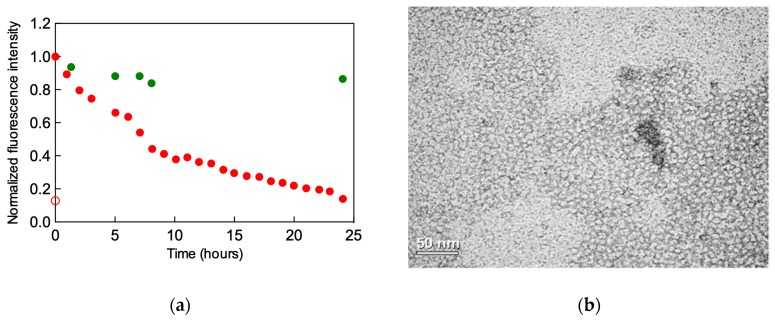

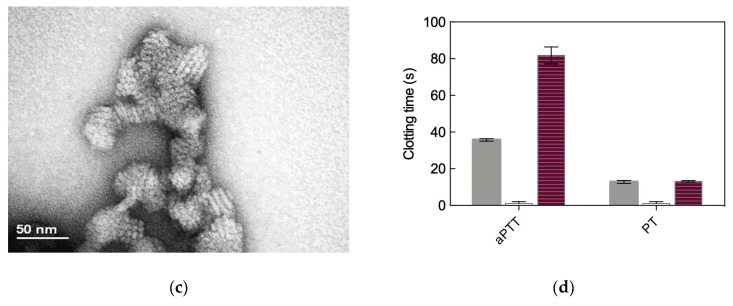
(**a**) Time-decay of the Nile Red normalized fluorescence intensity decay in buffer salted solution (pH = 7.4) in the presence of **C_22_G_1_** alone (red symbols) and **C_22_G_1_** and heparin (green symbols). The empty red dot at time = 0 is the light fluorescence observed in the presence of Nile Red alone in solution. TEM images of **C_22_G_1_** showing the spherical self-assembled nanomicelles in the absence (**b**) and in the presence (**c**) of heparin. (**d**) Clotting times measured in pure plasma (gray bars), in plasma + heparin (white bars), and in the presence of plasma + heparin + **C_22_G_1_** (gray-striped violet bars) by the aPTT and PT assays. Panels (**a**–**c**) adapted from [21] and [36], with the permission of The Royal Society of Chemistry.

Having ascertained the feasibility of our dendron degradation, we next verified that this process ultimately leads to the relevant self-assembled dendrimer disaggregation. To the scope, **C_22_G_1_** was dissolved into a buffer solution (pH = 7.4) at a final concentration above its CMC and in the presence of Nile Red. This dye is almost non-fluorescent in water and other polar solvents but undergoes fluorescence enhancement and large absorption and emission blue shifts in nonpolar environments. Accordingly, we measured the Nile Red fluorescence intensity over a 24-h time interval, obtaining the curve shown in red in Figure 5a. As it can be seen from this Figure, at the end of the assay the dye fluorescence has dropped to the same value measured in absence of the self-assembled dendrimers, allowing us to conclude that the degradation of the **C_22_G_1_** building blocks indeed resulted in the corresponding micelles disassembly. The same experiment performed with the additional presence of heparin led to the green curve in Figure 5a, from which the high fluorescence intensity still detected after 24 h suggested that, once bound to the polyanion, the self-assembled dendrimers are much more resistant to degradation and retain their stability. This indirect evidence was further supported by transmission electron microscopy (TEM) imaging, from which it is clear that, although obviously clustered around the heparin surface (Figure 5b), the spherical **C_22_G_1_** self-assembled nanostructures remain almost identical to those observed in the absence of the polyanion (Figure 5c) [21,36].

Therefore, under the perspective presented above, these results support the potential therapeutic use of the self-assembled dendrimers generated by **C_22_G_1_** in heparin rescue, in that excess nanomicelles with degrade and disaggregate over a relatively short time frame—a property that does not pertain to their covalent counterparts. Yet, when bound to heparin the **C_22_G_1_** nanoassemblies are more stable, hopefully allowing for the excretion of their intact heparin complexes.

The final step in the characterization of **C_22_G_1_** self-assembled dendrimers as potential protamine replacers was the determination of their ability to reverse the coagulation inhibition induced by heparin. As we know, blood coagulation is an exceedingly complex process [37] which, for the sake of brevity, can be simplified and condensed as follows. After any blood vessel endothelial injury, platelets (aka thrombocytes) quickly gather at the lesion site and create a cross-linked plug (primary hemostasis). This, in turn, activates the so-called coagulation cascade, with resultant fibrin deposition and linking (secondary hemostasis). Platelets retraction and inhibition, followed by wound repair, complete the process. For diagnostic/prognostic purposes, the clotting cascade is assumed to consist of two separate yet interactive pathways: The intrinsic pathway, activated by the external trauma that causes blood to leave the vascular system and involves factor VII; and the extrinsic pathway, which originates by the trauma within the vascular system, is activated by platelets, exposed endothelium, chemicals, or collagen, and involves factors XII, XI, IX, and VIII. Typically, the intrinsic route is quantified by the activated partial thromboplastin time (aPTT) assay, which measures the time required by the complex formed among various plasma clotting factors (called thromboplastin) to convert prothrombin to thrombin and, hence, generate the fibrin clot. The extrinsic pathway, on the other hand, is routinely monitored by the prothrombin clotting time (PT) assay, a one-stage test based upon on the time required for a fibrin clot to form after the addition of tissue factor (TF, aka called platelet tissue factor, factor III, or CD142, an integral transmembrane receptor for Factor VII/VIIa), phospholipid, and calcium to decalcified, platelet-poor plasma.

Accordingly, both aPTT and PT tests were performed to check the capacity of our **C_22_G_1_** self-assembled dendrimers to reverse the effect of heparin (Figure 5d). The experimental clotting time of pure plasma was equal to 35.7 s according to the intrinsic (aPTT) assay and to 12.8 s when measured by the PT assay. When heparin was added to plasma, the clotting time reduced to ~0, as expected from the anticoagulant activity exerted by the polyanion. On further addition of **C_22_G_1_** nanomicelles, however, although for some reasons still unknown the clotting time measured by the aPTT assay increased to 81.8 s, the PT assay yielded a clotting time of 13.1, very close to that of native plasma, supporting the concept that the self-assembled **C_22_G_1_** dendrimers are endowed of functional heparin reversal in the most biologically relevant environment.

In aggregate, all positive and innovative results obtained with the **C_22_G_1_** pseudo-dendrimers as potential protamine replacers in heparin-induced anti-coagulation inhibition strongly encouraged us to embark on further studies focused on further design/optimization of self-assembling amphiphilic dendron structures and related structure-activity relationships aimed at gaining a more fundamental understanding of heparin binding phenomena with the ultimate goal of contributing in paving the way of these nanotechnologically-driven compounds to pre-clinical investigations.

### 3.2. The Impact of Self-Assembled Dendrimer Morphologies on Heparin Binding

**Figure 6:**
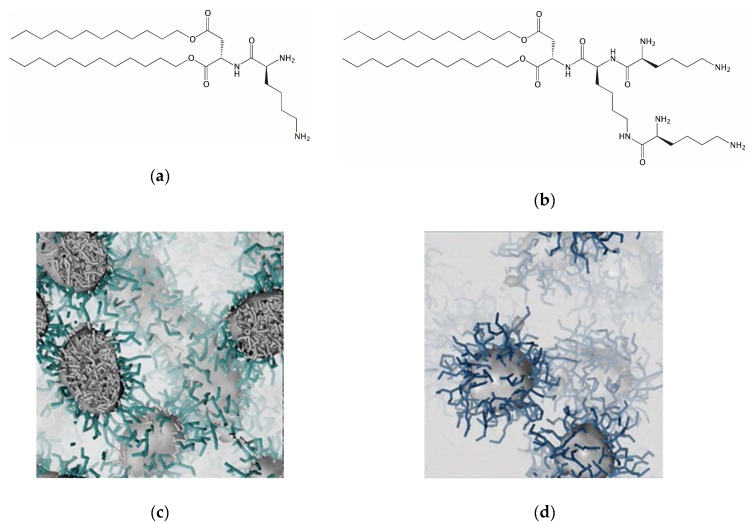
Chemical structure of the amphiphilic dendrons **L-G_1_** (**a**) and **L-G_2_** (**b**). Generation of worm-like (**c**) and spherical nanomicelles (**d**) upon self-assembly of **L-G_1_ and L-G_2_** under 150 mM NaCl and pH = 7.4 conditions, as obtained from multiscale molecular simulations. In panels (**c**) and (**d**) the common hydrophobic micellar core is evidenced as a gray-shaded surface (and gray sticks), while the hydrophilic shell is shown as forest green and steel blue sticks for **L-G_1_** (**c**) and **L-G_2_** (**d**), respectively. In both cases, the solvated salted environment is portrayed as a light gray field. Adapted from [26] with the permission of The Royal Society of Chemistry.

Having established the efficacy of the **C_22_G_1_** self-assembled dendrimers for coagulation control in terms of high-affinity, multivalent electrostatically-driven heparin binding and pharmaceutically-useful degradation, and disassembly profile, we decided to investigate the impact of amphiphilic dendron structural modifications on their self-assembled morphology and the related polyanion binding effect. Computer-assisted design based on multiscale molecular simulations ultimately yielded the two dendritic cationic compounds **(C_12_)_2_-L-Asp-L-Lys** and **(C_12_)_2_-L-Asp-L-Lys-(L-Lys)_2_** (hereafter called **L-G_1_** and **L-G_2_**, Figure 6a,b), which were predicted to self-assemble into worm-like cylindrical and spherical micelles, respectively (Figure 6b,c) [26,38].

After the synthesis of these two amphiphilic dendrons was carried out by peptide chemistry and protecting group methodologies, we first experimentally determined both their overall dimensions (by DLS), and self-assembly thermodynamics—via isothermal titration calorimetry (ITC). ITC is a straightforward, non-invasive, and highly-sensitive titration-based method that provides a complete and accurate thermodynamic description of different association processes (e.g., self-assembling and binding, just to name a few) in complex systems [39]. In line with the in silico results, for **L-G_2_**, DLS data confirmed the formation of spherical nanomicelles with average D_m_ = 6.7 ± 0.2 nm, and a positive surface charge, quantified by the zeta-potential value (ζ) of +29.6 ± 2.3 mV. Also, for **L-G_1_**, DLS supported the modeling view of its self-assembling in non-spherical, larger structures. In fact, the relevant data, fitted using an equivalent sphere that had the same average translational diffusion coefficient as the worm-like micelles, yielded substantially larger dimensions (D_m_ = 125 ± 10 nm) and higher ζ value (+73.2 ± 3.3 nm) for the corresponding nanoassemblies.

Next, ITC provided an extremely important insight into the thermodynamics of self-assembled dendrimer formation by these two new amphiphilic molecules. Indeed, from the experimental ITC titration curves the micellization enthalpy (ΔH_mic_) and CMC values could be directly determined. Next, the other thermodynamics parameters could be obtained as follows: Once each CMC value was known, the corresponding energy of micellization (ΔG_mic_) was calculated by the expression ΔG_mic_ = RT ln CMC, where R is the gas constant (8.31 × 10^−3^ kJ/mol K) and T is the absolute temperature. Finally, the change in entropy associated with micellization (TΔS_mic_) was estimated from the second law of thermodynamics by using the Gibbs–Helmholtz equation TΔS_mic_ = ΔH_mic_ − ΔG_mic_. According to the ITC results, the CMC and ∆G_mic_ values were equal to 58 µM and −34.13 kJ/mol for **L-G_1_** and to 13 µM and −38.03 kJ/mol for **L-G_2_**, respectively, suggesting that the latter amphiphilic dendron is more effective in terms of self-assembling thermodynamics. Further analysis of the ITC data yielded the corresponding values of ∆H_mic_ (−3.58 kJ/mol for **L-G_1_** and −2.40 kJ/mol for **L-G_2_**), and T∆S_mic_ (+30.55 kJ/mol for **L-G_1_** and +35.63 kJ/mol for **L-G_2_**, respectively). These data clearly indicated that the entropic contribution dominates the micellization process of these molecules in buffered solutions, with enthalpy playing a minor role (|TΔS_mic_|>>|ΔH_mic_|). Moreover, **L-G_2_** self-assembling is more favored on entropic grounds because not only does each small spherical micelle contains far fewer molecules with respect to the worm-like ones, but also a much larger number of nano-objects are formed.

#### Heparin Binding by **L-G_1_** and **L-G_2_** Self-Assembled Dendrimers: When Less Is More

The affinity of **L-G_1_** and **L-G_2_** micelles for heparin was initially investigated using the MB displacement assay in buffered solution at physiological pH (7.4) and ionic strength (150 mM NaCl). From the results shown in the upper part of Table 3 it appears evident that the small, spherical **L-G_2_** micelles are substantially more efficient heparin binders than the worm-like assemblies formed by **L-G_1_**. In our opinion, this result was somewhat counterintuitive, as we reasoned that the larger, charge-denser worm-like nano-objects should produce a greater number of stronger contacts with the polyanion than the smaller, less charged spherical ones.

**Figure 7:**
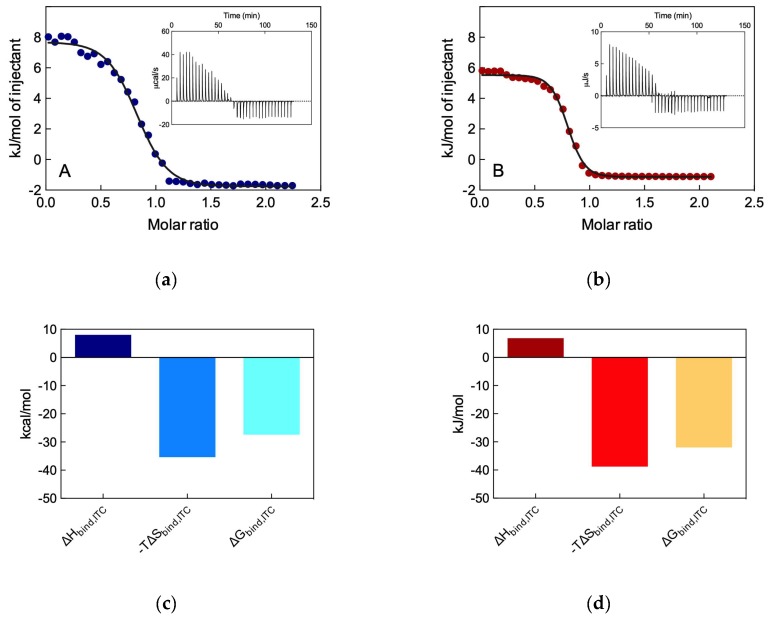
ITC isotherms for self-assembled dendrimers **L-G_1_** (**a**) and **L-G_2_** (**b**) heparin binding in buffered solutions at 150 mM NaCl. In both panels, ITC raw data are shown in the inserts while black curves are data fitting. Heparin binding thermodynamic parameters for **L-G_1_** (**c**) and **L-G_2_** (**d**), as obtained from the analysis of the corresponding ITC traces shown in panels **a** and **b**, respectively. Adapted from [26] with the permission of The Royal Society of Chemistry.

To verify the reliability of these unanticipated results, we performed again heparin binding experiments this time using ITC. In a way similar to that described for the determination of the micellization thermodynamics, during the binding experiment the ITC curves directly yield the binding enthalpy (∆H_bind,ITC_) and, upon data fitting with a suitable thermodynamic model, the binding constant K_b,ITC_ can be obtained. Once K_b_ in known, the free energy of binding ∆G_bind_ can be calculated using the relationship ∆G_bind,ITC_ = −RT ln K_b,ITC_, and the variation in entropy upon binding can again be derived T∆S_bind,ITC_ = ∆H_bind,ITC_ − ∆G_bind,ITC_. The relevant results are illustrated in Figure 7.

This Figure reveals that both self-assembled dendrimers bind heparin with positive (i.e., endothermic) ∆H_bind,ITC_ values equal to 8.03 ± 0.17 kJ/mol for **L-G_1_** and 6.82 ± 0.14 kJ/mol for **L-G_2_**, respectively. This unfavorable enthalpic contribution, however, is overcompensated by the highly positive entropic terms (T∆S_bind,ITC_ = +35.39 kJ/mol ± 0.60 kJ/mol and +38.80 kJ/mol ± 0.59 kJ/mol for **L-G_1_** and **L-G_2_**, respectively), mainly ascribable to the release, upon binding, of water molecules and counterions from the polycation/micelle contact surface into the bulk solvent. Notably, this effect is greater in the case of **L-G_2_**, as spherical micelles have much larger surface areas to desolvate with respect to worm-like ones. In aggregate, this results in an overall stronger heparin binding by the **L-G_2_** self-assembled dendrimers, with a ∆G_bind,ITC_ value of −31.98 ± 0.56 kJ/mol, 4.62 kJ/mol more favorable than the **L-G_1_**-based counterparts (∆G_bind,ITC_ = −27.36 ± 0.61 kJ/mol). From these data we then learned that small spherical self-assembled dendrimers, composed by few building blocks and characterized by a low surface charge are more effective heparin binder than the bigger, worm-like and charge-denser counterparts, i.e., less is more.

When tested for heparin affinity in a much more competitive and bio-relevant medium such as 100% human serum, interestingly enough the **L-G_2_** self-assembled dendrimers were adversely affected while the **L-G_1_** nanoassemblies practically retained their heparin binding ability, as seen in the lower part of Table 3. This suggests the idea that, in a challenging environment, spherical micelles are endowed with lower stability while nano-objects, which self-organize in other (e.g., worm-like) morphologies, not only can better sustain disaggregation but also can master multivalent binding strength at the self-assembled/polyanion interface more efficiently. However, even if the **L-G_2_** nanomicelles are subjected to this moderate reduction in polyanion binding in serum (the same mechanism leading to a decreased performance under similar conditions devised for the **C_22_G_1_** assemblies discussed above being at play also in this case), these spherical self-assembled dendrimers remain more efficient than the worm-like ones (CE_50_ = 0.85 ± 0.02 for **L-G_2_** and 1.15 ± 0.05 for **L-G_1_**, respectively, Table 3). Hence, less is still more.

**Figure A1:**
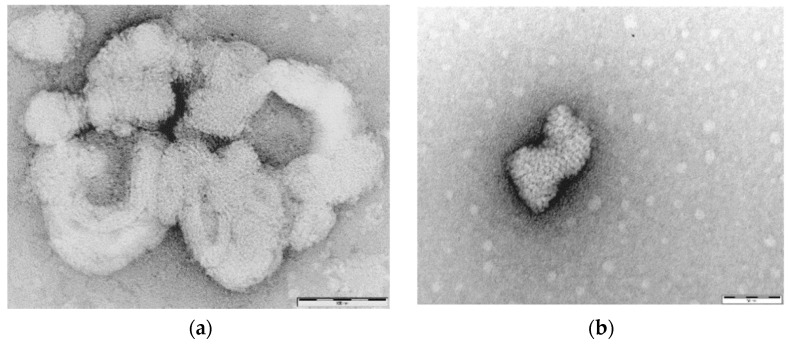
(**a**) TEM images of worm-like micelles of **L-G_1_** bound to heparin, scale bar = 100 nm. On drying, the worm-like micelle-heparin complex hierarchically assembles into larger aggregates. Worm like micelles can most clearly be visualized curved into ‘U shapes’ in the lower aggregates—multiple cylindrical micelles appear to be packed together in these regions. (**b**) TEM images of spherical micelles of **L-G_2_** bound to heparin, scale bar = 50 nm. Some free micelles can be seen within the image, but most have further aggregated hierarchically on binding to heparin and drying to yield a nanostructure which in this case is ca. 50 nm in diameter. Adapted from [26] with the permission of The Royal Society of Chemistry.

**Figure A2:**
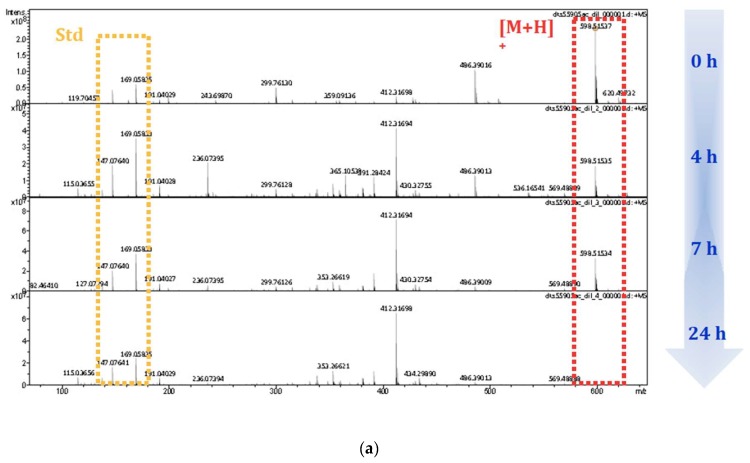

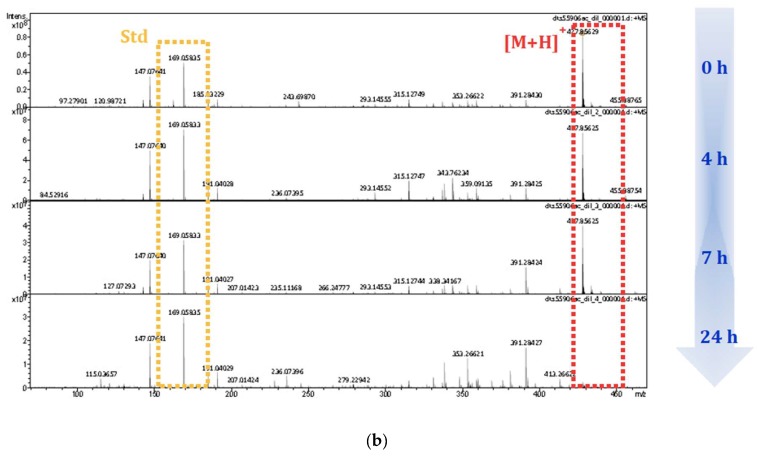
Electron spray mass spectra for the degradation of **L-G_1_** (**a**) and **L-G_2_** (**b**) self-assembled dendrimers dissolved in ammonium bicarbonate at pH = 7.4 TEM images of worm-like micelles of **L-G_1_** bound to heparin, scale bar = 100 nm. On drying, the worm-like micelle-heparin complex hierarchically assembles into larger aggregates. Worm like micelles can most clearly be visualized curved into ‘U shapes’ in the lower aggregates—multiple cylindrical micelles appear to be packed together in these regions. (**b**) TEM images of spherical micelles of **L-G_2_** bound to heparin, scale bar = 50 nm. Some free micelles can be seen within the image, but most have further aggregated hierarchically on binding to heparin and drying to yield a nanostructure which in this case is ca. 50 nm in diameter. Adapted from [26] with the permission of The Royal Society of Chemistry.

As for the case of the self-assembled nanomicelles formed by **C_22_-G_1_**, TEM imaging performed with and without heparin confirmed that both spherical and worm-like micelles were stable and, in the case of heparin, aggregate with the polyanion into larger, hierarchical structures, as shown in Figure A1 [26]. Also, the presence of the ester central linker in both **L-G_1_** and **L-G_2_** molecules (see Figure 6a,b) may also potentially subject the relevant nanostructures to degradation—and hence disassembly—under physiological conditions. Therefore, we employed again ESMS-based assays to probe this eventuality. Indeed, in utter analogy with what was observed for **C_22_G_1_**, both the spherical and the worm-like self-assembled dendrimers fully degraded via ester hydrolysis in 24 h (see Figure A2a,b), endowing these compounds with pharmaceutically useful degradation profile for heparin reversal—micelles administered in excess will degrade into isolated, non-active fragments. Nonetheless, although we further learned that different programmable nanoscale morphologies can offer a key alternative strategy to design and optimize new heparin binders, when comparing the overall performance of the **C_22_G_1_**, **L-G_1_**, and **L-G_2_** self-assembled dendrimers in binding this anionic polysaccharide, we realized that less is more indeed constitutes a new paradigm, since spherical nanomicelles still represent the most effective, efficient, and safe assemblies as potential protamine replacers for heparin reversal.

### 3.3. “Less Is More” Further Investigated

**Figure 8:**
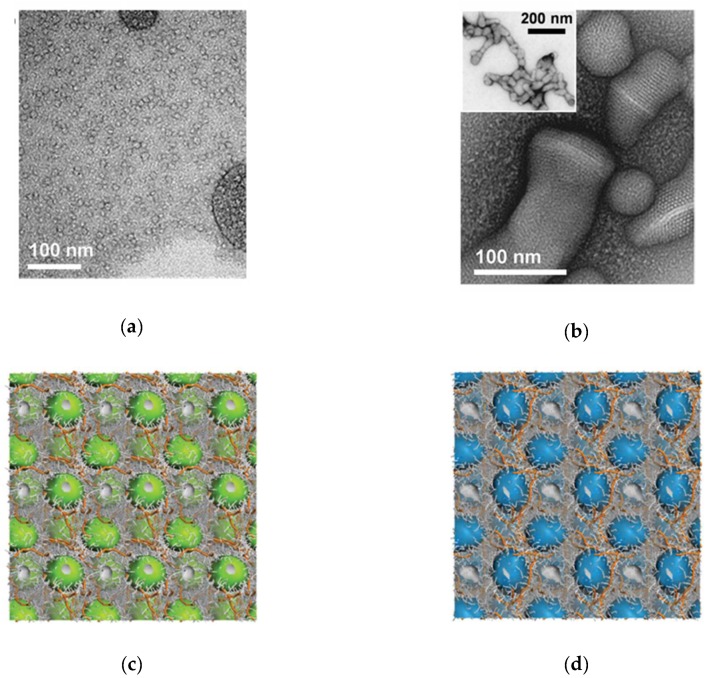
TEM images of **C_16_G_0_** self-assembled dendrons alone (**a**) and in the presence of heparin (**b**). In panel (**b**), the inset shows the full image indicating the overall nanoscale aggregate. Mesoscale molecular simulations images of **C_14_G_0_** (**c**) and **C_16_G_0_** (**d**) self-assembled dendrimers in the presence of heparin (heparin/binder ratio 1:2) in buffered solutions at 150 mM NaCl. The hydrophobic micellar core of is highlighted as green and blue surfaces, respectively, while the hydrophilic parts of each dendron type are shown as white sticks. Heparin chains are portrayed as orange rods, and the solvated environment is visualized as a continuous gray field. Adapted from [24] with the permission of The Royal Society of Chemistry.

We found the concept that small spherical micelles retain their shape while forming mesoscopic aggregate structure upon heparin binding quite intriguing. Accordingly, to investigate this aspect in more detail, we designed and optimized synthetically-simple, minimal self-assembling dendrons that could serve this purpose, ultimately ending up with “reduced versions” of **C_22_G_1_**, which is two molecules still featuring a single DAPMA polar head and a single apolar chain of 14 (**C_14_G_0_**) and 16 (**C_16_G_0_**) carbon atoms, respectively [24]. As per molecular design, in buffered solutions at physiologically ionic strength these two amphiphilic dendrons self-assembled into small, spherical nanostructures with high and positive ζ potentials (D_m_ = 5.8 ± 1.6 nm and ζ = + 41.3 ± 1.6 mV for **C_14_G_0_** and 6.2 ± 1.3 nm and + 51.7 ± 2.2 mV for **C_16_G_0_**, respectively), and showed good heparin binding ability (CE_50_ = 0.88 and 0.46 for **C_14_G_0_** and **C_16_G_0_**, respectively) yet inferior to those of C_22_G_1_, indicating that the structure this latter amphiphile was indeed optimized for binding the polyanion. However, given that both these simpler dendrons were endowed with the required characteristics (spherical micelle formation and heparin binding), we employed them to investigate the structure of the relevant heparin complexes starting from TEM imaging, as shown in Figure 8a,b for **C_16_G_0_** as an example (analogue images were obtained for the alternative **C_14_G**_0_-based system). These TEM observations, beside confirming the spherical nature of these self-assembled dendrimers, suggest that their stability is preserved, without disruption or reorganization, upon heparin binding with which they form strong electrostatic interactions. However, since we were aware that TEM images were collected on dried samples, we wondered whether the drying process might have somewhat forced the systems (and, by extensions, also the other self-assembled dendrimers previously discussed) to assume such morphology. Therefore, we first resorted again to molecular simulations to predict the self-assembly and spatial organization of these two amphiphiles in the presence of heparin in solution. The output of these simulations (Figure 8c,d) shows that not only both molecules self-assemble in small, stable spherical micelles in the presence of the polyanion but, perhaps even more important, these are not randomly dispersed into the heparin solution but adopt a highly-ordered, hierarchical nanoscale structure matching a face-centered (*fcc*) organization, with lattice constant value *a* equal to 8.1 nm and 8.6 nm for the **C_14_G_0_** and **C_16_G_0_** self-assembled dendrimers, respectively. The corresponding center-to-center distance ($a\sqrt{2}$) is equal to 5.7 nm in the case of the **C_14_G_0_** micelles and to 6.1 nm for the self-assembled **C_16_G_0_**, in excellent agreement with the D_m_ values measured by DLS (5.8 and 6.2 nm, respectively, see above).

**Figure 9:**
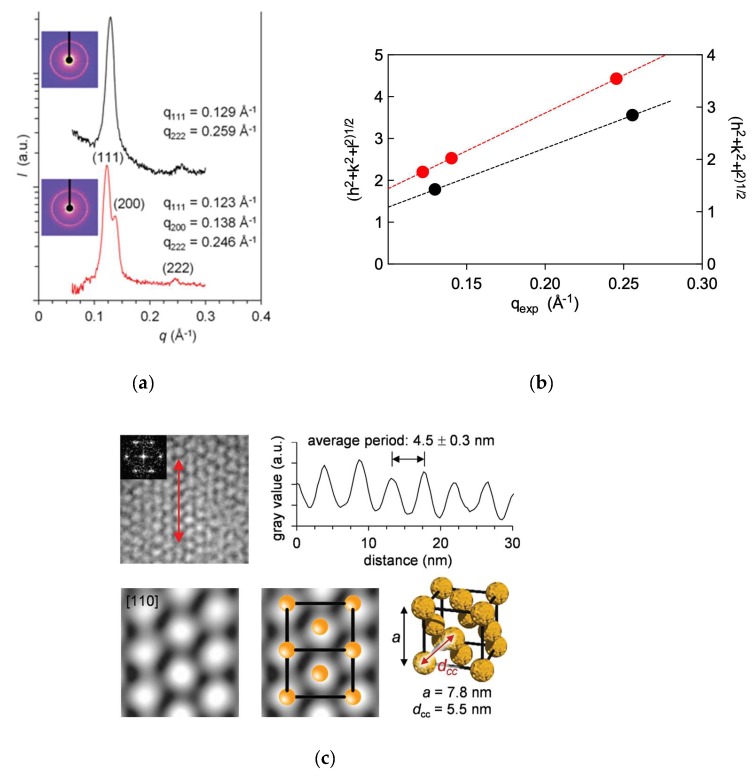
(**a**) Integrated SAXS curve measured from self-assembled dendrimers **C_14_G_0_** (black upper curve) and **C_16_G_0_** (red lower curve) in the presence of heparin. The insets show the corresponding 2D diffraction patterns. (**b**) Quadratic Miller indices of assigned reflections for face-center cubic (*fcc*) structures as function of measured *q*-vector positions for indexed peaks, related with heparin-bound **C_14_G_0_** (black symbols) and **C_16_-G_0_** micelles (red symbols). (**c**) (Top row) A crystalline area for **C_14_G_0_** (top left; inset: Fast Fourier transform) and a line profile analysis (top right) along the red double pointed arrow shown in the top left panel. (Bottom row) Filtered inverse Fourier transform from selected Fourier components for the **C_14_G_0_** (left), overlay of the image and the *fcc* unit cell (center), and model of the unit cell with key dimensions (right). The micelles are shown in yellow using a reduced D_m_ value for clarity. Adapted from [26] with the permission of The Royal Society of Chemistry.

With these comforting in silico indications at hand, we proceeded to an experimental verification via small angle X-ray scattering (SAXS). The relevant results are shown in Figure 9. In particular, for both self-assembled dendrimers/heparin supermolecular complexes, the bi-dimensional (2D) SAXS diffraction patterns are characterized by a Debye ring with a diffuse symmetric halo that does not present intensity differences (inserts in Figure 9a)—a distinctive feature of polycrystalline samples with isotropic orientation of multiple crystals [40].

As noted in Figure 9a, the positions of the diffraction peak for the **C_14_G_0_** micelles bound to heparin locate at values of the momentum transfer (aka scattering vector) *q* equal to 0.129 and 0.259 Å^−1^ which, assuming a *fcc* morphology, in terms of crystal plane reflections with Miller indices correspond to (*hkl*) = (111) and (222). The alternative, self-assembled dendrimers **C_16_G_0_** in complex with heparin showed 3 diffraction peaks, at *q* = 0.122, 0.138, and 0.246 Å^−1^, respectively. These, again using Miller indices notation for an *fcc* structure, correspond to (hkl) = (111), (200), and (222). By plotting the quadratic Miller indices against the measured q(*hkl*) values for both systems, the experimental lattice constant *a* $=2\pi \sqrt{h^{2}+k^{2}+l^{2}/q\left(hkl\right)}$ could be estimated by data linear fitting (Figure 9b) as 8.5 and 8.9 nm for the **C_14_G_0_** and **C_16_G_0_** systems, respectively. The corresponding experimental center-to-center distances $a\sqrt{2}$ were also calculated as 6.0 nm for the C_14_G_0_ micelles and 6.3 nm for the G_16_G_0_ assemblies, and all these values were in excellent agreement with those predicted by computer simulations (see above).

In a conclusive effort, experimental (SAXS) and computational data for both self-assembled dendrimers in complex with heparin were compared with the corresponding TEM images, as shown in Figure 9c. The top left panel in this Figure illustrates the crystal projection view along the [110] zone axis. The analysis of the linear profile over the crystal projection (red double pointed arrow) yielded an average period (*ap*) of 4.5 nm, corresponding to *fcc* lattice constant values of 7.8 and 8.0 nm for **C_14_G_0_** and **C_16_G_0_** nanoassemblies, respectively. These values agree with those derived from simulation and SAXS reported above, the minor reduction in the cell unit size being ascribable to the drying effect on the TEM grid. The Fast Fourier (FF) transforms of the crystalline area and the subsequent selected filtered inverse FF transforms yielded the representative image of the crystal cell (Figure 9c, bottom row, left). The *fcc* arrangement of the micelles was confirmed by superposing this image with the corresponding unit cell model (Figure 9c, bottom row, center). Finally, the micelle center-to-center distance values of 5.5 nm and 5.6 nm estimated for **C_14_G_0_** and **C_16_G_0_**, respectively (Figure 9c, bottom row, right), are in good agreement both with those obtained by computer simulations and with the micellar diameters estimated by DLS.

In summary, these results contributed for the first time to verify that even the “reduced version” of the amphiphilic dendron **C_22_G_1_**, that is the single-tail, small DAPMA-based **C_14_G_0_** and **C_16_G_0_** amphiphiles, are able to form very stable spherical micelles even when electrostatically bound to heparin, and that the presence of the polyanion induces the adoption of a highly regular, nanoscale hierarchical structure.

### 3.4. The Role of Dendron Head in Self-Assembled Dendrimers for Heparin Binding

As the two self-assembled dendrimers **C_14_G_0_** and **C_16_G_0_** proved to be excellent simple test systems, we next decided to use closely-related analogs to explore the eventual effect exerted by the hydrophilic portion on the formation and heparin binding performance of self-assembled dendrimers. Indeed, we reasoned that since heparin backbone is decorated with anionic sulfate and carboxylate residues, an eventual preferential polyanion binding by a given positive charge bearing ligand could enable the development of systems better optimized for the specific underlying clinical application.

**Figure 10:**
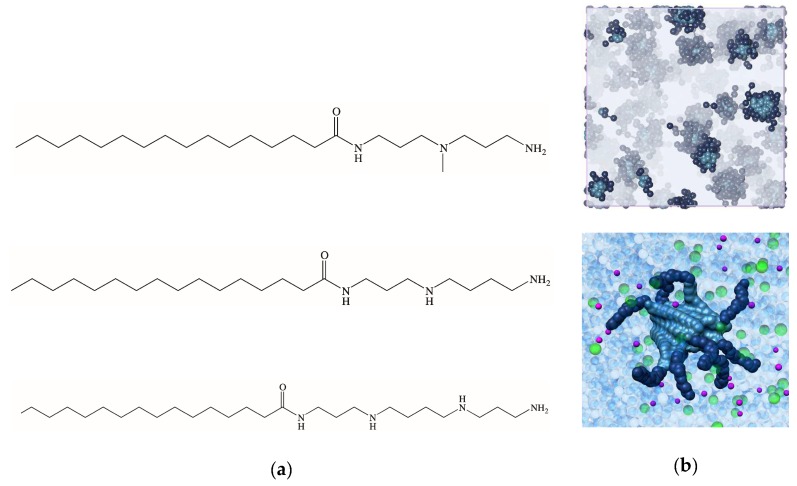
(**a**) Structures of **C_16_G_0_** (top), **C_16_SPD** (middle), and **C_16_SPM** (bottom) amphiphilic dendrons. At physiological pH, the former two dendrons bear 2 positive charges while **C_16_SPM** has a charge of +3. (**b**) Mesoscale (top) and atomistic (bottom) structures **C_16_SPM** self-assembled dendrimers. The hydrophobic micellar core is shown as steel blue spheres while the hydrophilic **SPM** residues are portrayed as navy blue spheres. In the top panel, the solvated environment is shown as a light gray field. In the bottom panel, water molecules are represented as transparent light blue spheres, with some Na^+^ and Cl^−^ ions shown as pink and green spheres, respectively. Adapted from [22] with the permission of The Royal Society of Chemistry.

Accordingly, starting from the best performing test system **C_16_G_0_**, we synthesized 2 further amphiphilic dendrons bearing the same C_16_-long hydrophobic chain and different hydrophilic heads—specifically spermidine (SPD) and spermine (SPM)—as shown in Figure 10a.

As for **C_16_G_0_** (see Section 3.2), multiscale molecular simulations carried out in a solvated environment containing 150 mM NaCl anticipated the aggregation of both new molecules in spherical micelles (Figure 10b), with D_m_ values of 6.3 ± 0.1 and 5.8 ± 0.2 nm for **C_16_SPD**, and **C_16_SPM**, respectively, and N_agg_ decreasing in the order (**C_16_G_0_** (16) >) **C_16_SPD** (13) > **C_16_SPM** (10). Evidently, the C_16_ alkyl chain is optimized for the **G_0_** head and is less effective in packing the longer (**SPD**) and/or more charged **SPM** ligands. As a consequence of the decrease in N_agg_, the predicted self-assembled dendrimers electrostatic potential Ψ also decreased in the same order (172.4, 153.3, and 144.6 mV), resulting in the corresponding z-potential values of 50.2, 45.1, and 41.8 mV, respectively. DLS confirmed the modeling data, with D_m_ and ζ-potential values of 6.6 ± 0.2 nm and +44.0 ± 1.7 mV for **C_16_SPD** and 6.2 ± 0.1 nm and +40.5 ± 0.9 mV for **C_16_SPM**, respectively.

The ability of **C_16_SPD** and **C_16_SPM** to bind heparin was tested via the MB assay [33,34]. Compared to **C_16_G_0_** (CE_50_ = 0.69, Section 3.2), the two new nanosystems were both more efficient, **C_16_SPD** being the best polyanion binder (CE_50_ = 0.34) followed by **C_16_SPM** (CE_50_ = 0.49). These results were confirmed by direct binding measurement performed via isothermal titration calorimetry. ITC data, expressed in terms of free energy of binding ∆G_bind_, were indeed in broad agreement with those obtained from MB displacement (∆G_bind_ = −2.2 kJ/mol for **C_16_G_0_** and −4.9 kJ/mol for both **C_16_SPD** and **C_16_SPM**), **C_16_SPD** still being the most effective heparin binder especially when the ∆G_bind_ values were normalized per dendron charge (−2.45 kJ/mol for **C_16_SPD** (+2), −1.63 kJ/mol for **C_16_SPM** (+3), and −1.1 kJ/mol for **C_16_G_0_** (+2), respectively).

**Figure 11:**
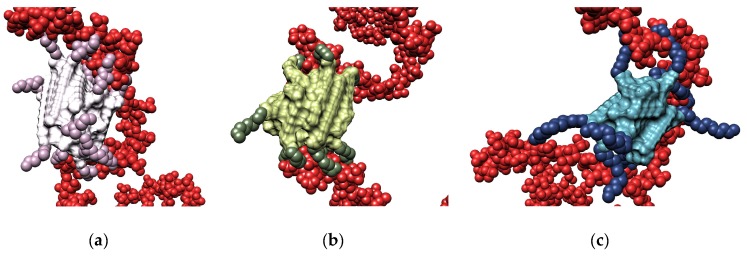
Equilibrated atomistic MD simulation snapshots of heparin in complex with the self-assembled dendrimer **C_16_G_0_** (**a**), **C_16_SPD** (**b**), and **C_16_SPM** (**c**). In all panels, heparin is in a red sphere representation, while the self-assembled dendrimer micelles are represented in colored spheres, as follows: (**a**) **C_16_**, light gray; **G_0_**, plum; (**b**) **C_16_**, lime green; **SPD**, forest green; (**c**) **C_16_**, steel blue; **SPM**, navy blue. Hydrogen atoms, water molecules, ions and counterions are omitted for clarity. Adapted from [22] with the permission of The Royal Society of Chemistry.

Insights into the molecular reasons underlying this trend were derived again from MD simulations (Figure 11).

The analysis of each heparin/self-assembled dendrimer complex MD trajectory revealed that the micelles formed by **C_16_SPD** productively engaged 12 out of 13 available dendrons in polyanion binding, resulting in a charge-normalized, per-effective-residue free energy of binding ∆G* of −14.98 kJ/mol. On the other hand, the other two nanostructures exploited only 9/16 (**C_16_G_0_**) and 6/10 (**C_16_SPM**) residues in productive binding, resulting in lower ∆G* values of −8.65 and −11.97 kJ/mol, respectively. The in silico data were thus in agreement with the trend exhibited by the corresponding experimental CE_50_/ITC values, that is the affinity of the three self-assembled dendrimers for heparin decreases in the order **C_16_SPD** > **C_16_SPM** > **C_16_G_0_**. To get further details on the heparin binding by these three nanosystems, we reconsidered ITC data, and precisely the deconvolution of ∆G_bind_ into its enthalpic (∆H) and entropic (T∆S) components. These data revealed that the **C_16_SPD** self-assembled dendrimer attains the most favorable enthalpic contribution upon adapting and subsequently optimizing its interaction with the polyanion (∆H = −4.9 kJ/mol) with respect to the other two nanomicelles, for which ∆H = −1.9 kJ/mol (**C_16_G_0_**) and −2.6 kJ/mol (**C_16_SPM**), respectively. Notwithstanding the fact that the entropic penalty for the self-assembled dendrimer **C_16_SPD** was not the best in the series (T∆S = 0.0, 0.3, and 2.3 kJ/mol for **C_16_SPD**, **C_16_G_0_**, and **C_16_SPM**, respectively), the overall binding in all cases preserved its enthalpy-driven nature, confirming the heparin best binding properties of **C_16_SPD**.

The lesson we learnt from this combined study was that changing both the length (**C_16_G_0_** < **C_16_SPD** < **SPM**) and/or the charge (+2, +2, +3) of the amphiphilic dendron polar heads had a moderate effect on the affinity of the respective self-assembled dendrimers on heparin binding (CE_50_ = 0.69, 0.34, and 0.49 for **C_16_G_0_**, **C_16_SPD**, and **C_16_SPM**). At the same time, we confirmed that in these interactions the electrostatic ion-ion binding depends not only on the heparin and/or micelle charge densities but also from the polyanion and the self-assembled dendrimers structural details. Yet, given the higher CMC values of **C_16_SPD** (51 µM) with respect to **C_16_G_0_** (40 µM), coupled with its slightly lower water solubility and in any case a heparin binding affinity lower that the “lead” compound **C_22_G_1_** (CMC = 4 µM, CE_50_ = 0.28, Section 3.1), we decided to employed again the simpler test amphiphilic molecule **C_16_G_0_** in further studies, as described below.

### 3.5. The Role of Dendron Tail in Self-Assembled Dendrimers for Heparin Binding

**Figure 12:**
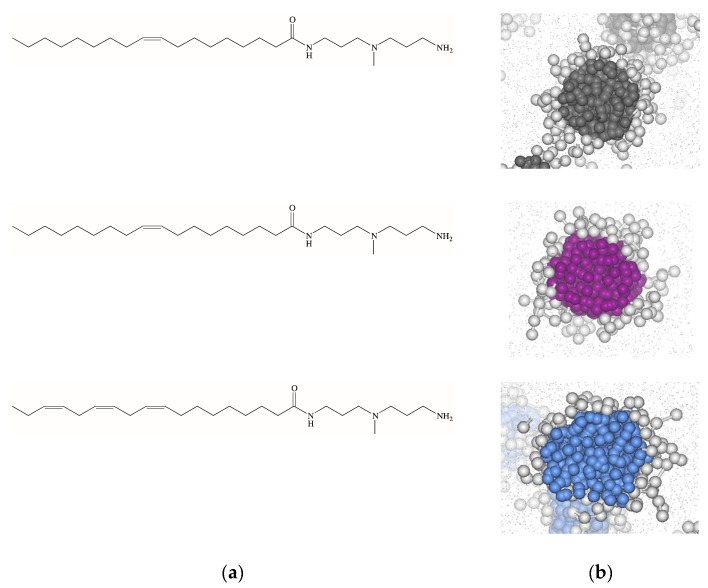
(**a**) Structures of the amphiphilic **C_18_G_0_** dendrons bearing 1 (**C_18,1_G_0_**, top), 2 (**C_18,2_G_0_**, middle), and 3 (**C_18,3_G_0_**, bottom) *cis*-alkene groups, respectively. (**b**) Mesoscale structures of the spherical micelles of the self-assembled dendrimer **C_18,1_G_0_** (top, left), **C_18,2_G_0_** (top, right), and **C_18,3_G_0_** (bottom). The hydrophobic micellar core is shown in color while the hydrophilic DAPMA portion is portrayed in light gray. Solvent and ions are represented by a dotted field. Adapted from [25] with the permission of John Wiley & Sons.

As the self-assembled dendrimers **C_14_G_0_** and **C_16_G_0_** proved to be an excellent simple test system, we next decided to use two closely-related analogs to explore the eventual effect exerted by the hydrophobic unit rigidity on their self-aggregation properties and the related heparin binding. To the purpose, we synthesized three new molecules still based on the polar DAPMA (G_0_) head yet this time appended to a C_18_-long carbon chain bearing 1, 2, or 3 unsaturations, respectively (see Figure 12a). The reason for using a C_18_ instead of the C_16_ fragment employed in the study discussed above was exclusively related to the possibility of incorporating up to 3 double bonds in the hydrophobic moiety still preserving the same, flexible, 7-carbon-long segment within the chain.

Multiscale computer simulations initially predicted all these three compounds to form spherical self-assembled dendrimers (Figure 12b) with micellar diameters, N_agg_ and ζ-potential values increasing with increasing number of unsaturated bonds along the apolar chain although, once two double bonds were introduced in the apolar tail, the addition of a third alkene moiety had relatively little additional effect (Table 4). The subsequent synthesis of these three amphiphilic dendrons and the experimental characterization of the relevant self-assembled forms fully confirmed the in silico data, as also shown in Table 4.

**Figure 13:**
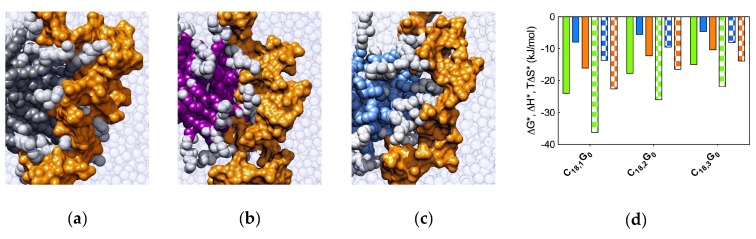
Zoomed view of the images extracted from the equilibrated portion of the MD simulations for self-assembled dendrimers **C_18,1_G_0_** (**a**), **C_18,2_G_0_** (**b**), and **C_18,3_G_0_** (**c**) in complex with heparin. In all panel, micelles are colored as in Figure 12b, heparin is portrayed by its orange van der Waals surface, while water, ions and counterions are shown as transparent spheres. (**d**) Per-residue effective free energy (∆G* = ∆G_bind,eff_/N_eff_, orange), enthalpy (∆H* = ∆H_bind,eff_/N_eff_, green) and entropy (T∆S* = ∆S_bind,eff_/N_eff_, blue) of binding for the three self-assembled dendrimers in panels (**a**–**c**) (solid bars) and for the heparin sugars complexed with each of these three self-assembled dendrimers (patterned bars). Adapted from [25], with permission of John Wiley & Sons.

When challenged for heparin binding using the MB assay [33,34], the ability of these nanoassemblies to complex the polyanion was found to decrease with increasing unsaturation, i.e., CE_50_ = 0.8 ± 0.5, 1.8 ± 01, and 2.3 ± 0.2 for **C_18,1_G_0_**, **C_18,2_G_0_**, and **C_18,3_G_0_**, respectively. As the difference in these CE_50_ values were well beyond the related uncertainties, we could conclude that the progressive stiffening of the dendron tails has a significant impact on their polysaccharide binding. To unveil the molecular reasons underlying this behavior, we resorted again to computer-based simulations. Figure 13a–c shows three equilibrated snapshots extracted from the corresponding MD simulations of the self-assembled dendrons in complex with heparin. These images clearly show that, in passing from 1 to 3 unsaturations in the hydrophobic portion of the self-assembling dendrons the number of positively-charged terminal groups in productive contact with the polyanion progressively decreases. In quantitative terms, this translates into N_eff_ values of 19, 15, and 13 for **C_18,1_G_0_**, **C_18,2_G_0_**, and **C_18,3_G_0_**, respectively.

From the energetic standpoint, from the solid bars in Figure 13d we see that, when heparin binding is considered from the perspective of each single micellar monomer, the self-assembling dendron bearing the most flexible tail (i.e., **C_18,1_G_0_**) is endowed with the most favorable enthalpic term (∆H* = −24.02 kJ/mol) which, by overcompensating the unfavorable entropic contribution (T∆S* = −7.92 kJ/mol), ultimately leads to a quite favorable value of ∆G* (−16.10 kJ/mol). As the number of double bonds increases and, as reported above, N_eff_ decreases, the relevant parameters of binding thermodynamics follow the same trend. Accordingly, for **C_18,2_G_0_** ∆H* = −17.76 kJ/mol, T∆S* = −5.55 kJ/mol, and ∆G* = −12.21 kJ/mol, while for **C_18,3_G_0_** ∆H* = −14.98 kJ/mol, T∆S* = −4.63 kJ/mol, and ∆G* = −10.35 kJ/mol, respectively. Of note, increasing the hydrocarbon chain stiffness brings about a small beneficial effect in entropic terms, as the molecules suffer less entropic penalties upon binding; yet, as at the same time, due to increased rigidity, the micelle monomers are less able to adapt their conformation for optimal electrostatic polyanion binding, and the enthalpic component is less effective in driving the intermolecular interactions. As a global result, the computational affinity ranking for the self-assembled dendrimers follows the same trend of their experimental counterpart, ie., **C_18,1_G_0_** > **C_18,2_G_0_** > **C_18,3_G_0_**. When the same analysis was applied from the viewpoint of heparin sugars, the variations of the three binding components (patterned bars in Figure 13d) paralleled those experienced by each micellar dendron. Accordingly, in binding the micelles generated by the most flexible dendron (**C_18,1_G_0_**) the polyanion can compensate a higher entropic penalty by gaining a substantially more favorable enthalpic contribution on its own, while it progressively adapts the enthalpy/entropy ratio on increasing micellar rigidity.

As the final step in this investigation, we experimentally evaluated the performance of these three self-assembled dendrimers in binding heparin in the presence of human serum. Although, as expected, this more competitive condition had an adverse effect on the micelle/polyanion interaction, the order in affinity was preserved, the corresponding CE_50_ values being 133 ± 10, 182 ± 15, and 166 ± 2 for **C_18,1_G_0_**, **C_18,2_G_0_**, and **C_18,3_G_0_**, respectively. Importantly, these values are still substantially lower than those pertaining to our “lead” self-assembled dendrimer **C_22_G_1_**.

Thus, the lesson we learned from this study is that, even though the molecular structural differences in these new three self-assembling dendrons are buried in the hydrophobic cores of the micelles they generate, with a mechanisms prototypical of dendrimeric materials these characteristics are transmitted through the entire nanoobjects, ultimately resulting into significantly different polyanion binding preferences, with heparin—an adaptive polyanion—being more affine to the micelles constituted by the most flexible monomers.

### 3.6. **CholSG_1_**: The Self-Assembled Dendrimer with Enhanced Stability in Human Serum

All the studies summarized thus far confirmed **C_22_G_1_** as the most effective amphiphilic dendron, which self-assembles into spherical micelles at very low concentration and is able to bind heparin with very high affinity (CE_50_ = 0.28)—decidedly higher than protamine (EC_50_ = 0.52)—in buffered solutions at physiological ionic strength (Section 3.1.1). However, the **C_22_G_1_** self-assembled dendrimers show some weakness when tested in a more clinically-relevant environment such as human serum (CE_50_ = 0.96), underperforming with respect to the current clinical heparin antidote (CE_50_ = 0.76, Table 1). In this respect, we speculated that kinetic micellar instability and the subsequent interaction of the alkyl chain with the most abundant serum protein (i.e., human serum albumin, HSA) could lead to **C_22_G_1_** micelles disassembly and, ultimately, loss of heparin binding.

**Figure 14:**
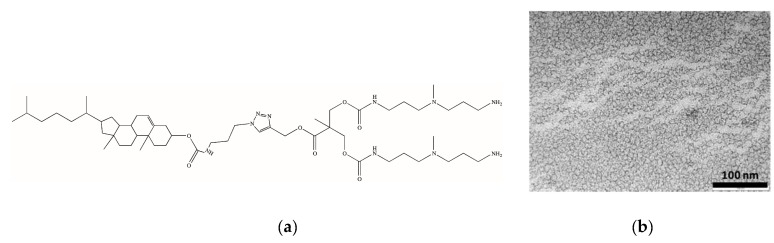
(**a**) Structure of the amphiphilic dendron **CholSG_1_**. (**b**) TEM images of the spherical micelles obtained upon self-assembling of **CholSG_1_**. Adapted from [29] with the permission of The Royal Society of Chemistry.

To verify this hypothesis, we decided to synthesize a new amphiphilic dendron still based on the **G_1_** head but with the incorporation of cholesterol (**Chol**) as the hydrophobic unit, since we previously observed good performance of cholesterol-based self-assembled dendrons in DNA binding and delivery [13,14]. Thus, starting from the structure of **C_22_G_1_**, we designed **CholSG_1_** (Figure 14a), in which a small spacer (**S**) between **Chol** and the triazole ring was inserted to minimize steric hindrance between dendron head and tail and to allow for some conformational mobility [29]. CholSG1 was readily synthesized by modifying **Chol** with an azide group, followed by conjugation with the alkyne-modified G1 head via click chemistry.

ITC measurements of **CholSG_1_** micellization gave a CMC of 8.1 µM, slightly higher than **C_22_G_1_** yet still below 10 µM, a value suitable for the foreseen application of these self-assembled dendrimers as heparin rescue. TEM images of the **CholSG_1_** nanoassemblies confirmed the formation of small spherical micelles (Figure 14b), for which DLS gave a mean average diameter of 7.5 ± 0.2 nm.

#### Heparin Binding of **CholSG_1_** in Buffer at Physiological Ionic Strength and in Human Serum

Both MB displacement assays and ITC measurements were initially conducted in buffer solution at 150 mM NaCl to evaluate heparin binding for the new **CholSG_1_** self-assembled dendrimer and to compare it with that of **C_22_G_1_** and, especially, protamine. Somewhat disappointedly, these new nanoassemblies were less effective (EC_50_ = 0.79 ± 0.02) than the other two reference compounds (EC_50_ = 0.28 and 0.52 for **C_22_G_1_** and protamine, respectively, Table 1), although TEM images of the **CholSG_1_** micelles in complex with heparin were utterly similar to those seen for **C_22_G_1_** under the same conditions (Figure 5c), showing no micellar morphology alteration or disaggregation upon polyanion binding. ITC experiments confirmed the results from the MB assays, in that the relevant ∆G_bind_ values were found to be equal to −30.85 kJ/mol for **CholSG_1_** vs. the slightly more favorable value of −32.73 kJ/mol observed for **C_22_G_1_**. However, for both self-assembled dendrimers the polyanion binding was enthalpically-driven, due to the substantial electrostatic nature of the underlying intermolecular interactions. So, ∆H_bind_ was equal to −21.18 and −20 71 kJ/mol for **C_22_G_1_** and **CholSG_1_**, respectively. Interestingly, in this case the entropic contribution to ∆G_bind_ was also substantially favorable (due to the release of water molecules and ions into the bulk solvent upon complex formation), with T∆S_bind_ values of 11.55 kJ and −10.14 kJ/mol for the dendrons bearing the linear alkyl segment and the **Chol** segment as the hydrophobic portion, respectively.

Notwithstanding this slight underperformance of the new self-assembled dendrimers **CholSG_1_**, we decided to challenge them for heparin binding in 100% serum. Pleasingly, the MB assay returned for them a CE_50_ value of 0.69 ± 0.07 (corresponding to a dose of 0.63 mg/100 heparin IU), which is sensibly lower than that obtained for both **C_22_G_1_** and protamine (0.96 and 0.79, respectively, Table 1). These good results led us back to the original hypothesis that the presence of a linear hydrocarbon chain in the **C_22_G_1_** dendron may results in micelles more prone to degradation by the action of serum proteins (in particular HSA, the most abundant serum protein with high affinity for alkyl-bearing compounds), whilst the nanostructures arising from the self-assembling of **CholSG_1_** might be less subjected to this disruptive protein action and, as such, can perform better in the more physiologically environment of 100% human serum.

**Figure 15:**
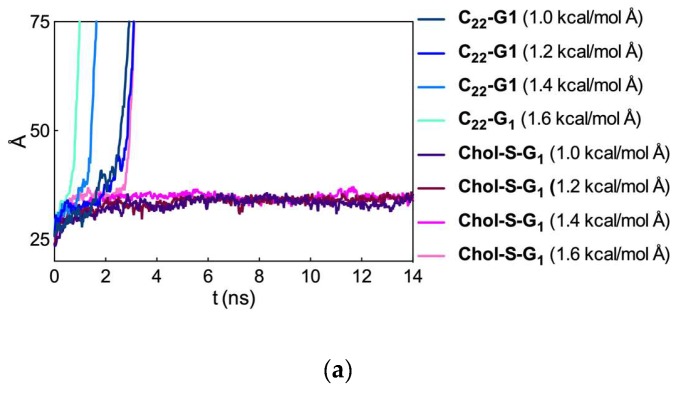

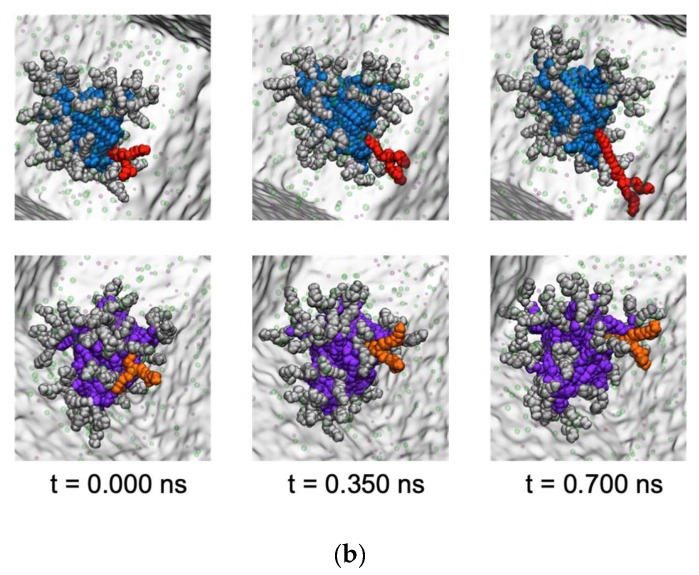
(**a**) Distance between the center of mass (COM) of the **C_22_G_1_** and the **CholSG_1_** self-assembled dendrimers and the COM of the selected dendron subjected to the different pullover forces (labelled on the left) during the corresponding CF-SMD simulations. (**b**) CF-SMD simulation snapshots of a micelle of the **C_22_G_1_** (upper panel) and the **CholSG_1_** self-assembled dendrimers TEM experiencing a pullout force of 1.6 kcal/mol Å taken at the same three representative times along the respective simulation trajectories. In both panels, the dendrons in each micelle are shown by their van der Waals surface, colored as follows: Hydrophobic cores, blue (**C_22_G_1_**) and purple (**CholSG_1_**); common hydrophilic head, gray; pullout monomers, red (**C_22_G_1_**) and orange (**CholSG_1_**). Na^+^ and Cl^−^ ions are shown as pink and green transparent spheres, while water is represented by a transparent light gray field. Adapted from [29] with the permission of The Royal Society of Chemistry.

To further confirm this concept, we performed two additional investigations, based on ITC experiments and computer simulations, respectively. The former, in which heparin binding to the **CholSG_1_** and **C_22_G_1_** micelles was carried out in the presence of 500 µM HSA, confirmed the results from the MB assay. In fact, while the serum protein did not significantly interfere with the **CholSG_1_**/heparin complexation (∆G_bind_ = −30.72 kJ/mol, ∆H_bind_ = −20.35 kJ/mol, and T∆S_bind_ = 10.37 kJ/mol), the binding of **C_22_G_1_** to the polyanion was appreciably affected by the presence of HSA in solution, resulting in decidedly less favored thermodynamics parameters (∆G_bind_ = −29.56 kJ/mol, ∆H_bind_ = −0.03 kJ/mol, and T∆S_bind_ = 9.53 kJ/mol). To gain further insights in to the eventual different kinetic stability of the two micelle types, we resorted to computer-based constant-force stirred molecular dynamics (CF-SMD) simulations. In detail, we evaluated the ability of the **CholSG_1_** and **C_22_G_1_** self-assembled dendrimers to withstand a force applied to pull out one of their respective dendron components. As shown in Figure 15a, the lower stability of the **C_22_G_1_** micelles was indeed confirmed by these in silico experiments, since even at the smallest pullout force applied (i.e., 1.0 kcal/mol Å) a **C_22_G_1_** dendron could be extracted from the relevant self-assembled dendrimer already during the early stages of the corresponding CF-SMD simulation. On the contrary, a substantially higher force (1.6 kcal/mol Å) was required to pullout a monomer from the **CholSG_1_** micelles. These quantitative observations are well represented by the corresponding images taken along the CF-SMD trajectories shown in Figure 15b.

As seen from Figure 15b, the **C_22_G_1_** dendron selected for pullout is completely extracted from its micelle already after only 0.7 ns of CF-SMD simulation (upper panel) while at the same time, the **CholSG_1_** alternative is still well inserted within the corresponding micellar structure. These results clearly indicate that the self-assembled dendrimers based on **Chol** as the hydrophobic unit are endowed with a substantially greater kinetic stability with respect to those featuring the linear C_22_- alkyl chain, thereby supporting the initial design hypothesis.

These results, coupled with further data showing that the cholesterol-based amphiphilic dendrons were endowed with significantly lower toxic effects than **C_22_G_1_** in a wide range of concentrations when assayed in a human hepatoblastoma cell line, make the **CholSG_1_** the new lead compound for further translation study as a potential clinical protamine replacer.

## 4. Conclusions

This review presents a collection of coupled experimental/computational studies taken from our own experience in the field of self-assembled dendrimers for heparin binding. These studies emphasize both the potentiality played by this hybrid methodology to the design, synthesis and development of possible protamine replacers in biomedical applications, and the obstacles this field has still overcome before these molecules can be translated into nanomedicines available in clinical settings. To date, reliable multiscale molecular simulations may be easier to perform than experiments. Accordingly, the synergist action of computer modeling and dedicated experiments can dramatically help in reducing the time and costs of the pre- and post-development stages of nanomedicines.

Under this perspective, we started the quest for possible protamine antidotes to be used for heparin anticoagulant activity reversal during chirurgical and other medical practices from covalent PAMAM dendrimers. The results from this investigation identified the EDA-core G_2_ PAMAM dendrimer as the preferred heparin binder both in buffered saline solution and in human plasma. Nonetheless, as a substantially more degradable, less expensive and easier-to produce new molecular entity could be more amenable for successive GMP production, we decided to exploit self-assembly to fabricate biologically-active nanosystems from simple low-molecular-weight building blocks. Efforts in this respect lead us to the design and synthesis of a plethora of amphiphilic dendrons differing in their polar, apolar, or both components. Collectively, the investigation of all these systems led us to gain a wealth of information about the role played by the nature of the dendron components both in their self-assembling structures and characteristics, and in their affinity for heparin in physiologically relevant environment such as 100% human serum, which will be exploited in our future work in this highly challenging sector of current nanomedicine.

## Figures and Tables

**Table 1:** Heparin binding data for **C_22_G_1_**, protamine, and the covalent G_2_ PAMAM dendrimer in 150 mM NaCl buffered solution (10 mM Tris-HCl, pH = 7.4) and in 100% human serum as obtained from the MB displacement assay. Adapted from [21], with the permission of The Royal Society of Chemistry. Data for protamine and G_2_ PAMAM—also shown in Table A1 for high salt conditions and reported in the main text for serum conditions—are taken from [33].

Heparin Binder	EC_50_ (µM)	CE_50_	Dose (mg/100 Heparin IU)
150 nM NaCl buffered solution			
**C_22_G_1_**	7.50 ± 1.22	0.28 ± 0.05	0.23 ± 0.04
protamine	2.34 ± 0.23	0.52 ± 0.05	0.32 ± 0.03
G_2_ PAMAM	2.55 ± 0.32	0.38 ± 0.04	0.25 ± 0.03
100% human serum, 10 mM Tris-HCl			
**C_22_G_1_**	29.90 ± 1.60	0.96 ± 0.06	0.79 ± 0.05
protamine	3.51 ± 0.12	0.79 ± 0.02	0.49 ± 0.02
G_2_ PAMAM	2.15 ± 0.05	0.32 ± 0.01	0.21 ± 0.01

**Table 2:** Effective free energy of binding (∆G_bind,eff_), number of effective protamine /dendrimer positive charges (N_eff_), and effective-charge-normalized free energy of binding (∆G_bind,eff_/N_eff_) for C22G1 self-assembled dendrimers, protamine and the covalent G2 PAMAM dendrimers in complex with heparin under 150 mM NaCl conditions as derived from atomistic MD simulations. Adapted from [33] with the permission of The Royal Society of Chemistry. Data for protamine and G_2_ PAMAM are taken from Table A2 [33].

Heparin Binder	N_eff_	∆G_bind,eff_ (kcal/mol)	∆G_bind,eff_/N_eff_ (kcal/mol)
**C_22_G_1_**	32 ± 1	−65.0 ± 0.05	−2.03 ± 0.08
protamine	12 ± 1	−3.96 ± 0.41	−0.33 ± 0.04
G_2_ PAMAM	13 ± 1	−16.9 ± 0.5	−1.30 ± 0.11

**Table 3:** Heparin binding data for the self-assembled dendrimers **L-G_1_** and **L-G_2_** in 150 mM NaCl buffered solution (10 mM Tris-HCl, pH = 7.4) and in 100% human serum as obtained from the MB displacement assay. Adapted from [26], with the permission of The Royal Society of Chemistry.

Heparin Binder	EC_50_ (µM)	CE_50_
150 nM NaCl buffered solution		
**L-G_1_**	59.9 ± 11.3	1.11 ± 0.21
**L-G_2_**	13.8 ± 0.70	0.51 ± 0.05
100% human serum, 10 mM Tris-HCl		
**L-G_1_**	61.9 ± 2.60	1.15 ± 0.05
**L-G_2_**	23.1 ± 0.50	0.85 ± 0.02

**Table 4:** Computational and experimental characterization of the self-assembled dendrimers formed by the three dendrons featuring different degrees of unsaturation in their hydrophobic part in 150 mM NaCl buffered solution (10 mM Tris-HCl, pH = 7.4). Adapted from [25], with the permission of John Wiley & Sons.

Self-Assembled Dendrimer	D_m,comp_ (nM)	D_m,exp_ (nM)	N_agg_ (-)	ζ_comp_ (mV)	ζ_exp_ (mV)	CMC (µM)
**C_18,1_G_0_**	5.4 ± 0.4	5.2 ± 0.5	28 ± 2	+63.2	+64.1 ± 0.6	42 ± 3
**C_18,2_G_0_**	6.2 ± 0.2	6.4 ± 0.4	32 ± 1	+73.4	+72.9 ± 3.7	82 ± 2
**C_18,3_G_0_**	7.2 ± 0.2	7.6 ± 0.3	35 ± 1	+75.2	+72.9 ± 2.5	78 ± 10

## Appendix A

**Table A1:** EC_50_ and CE_50_ values for protamine and for ethylenediamine-based covalent PAMAM dendrimers (G_0_-G_6_) as heparin binders. Values were obtained by fitting the data in Figure 2a. The last column shows the relevant dosage, i.e., the mass of binder required to bind 100 international units (IU) of heparin (i.e., the clinical standard. Adapted from [33] with the permission of The Royal Society of Chemistry.

Heparin Binder	Charge	EC_50_ (µM)	CE_50_	Dose (mg Binder/100 Heparin IU)
Protamine	+24	2.34 ± 0.23	0.52 ± 0.05	0.32 ± 0.03
G_0_	+4	too weak	too weak	too weak
G_1_	+8	10.10 ± 0.32	0.75 ± 0.02	0.44 ± 0.01
G_2_	+16	2.55 ± 0.32	0.38 ± 0.04	0.25 ± 0.03
G_3_	+32	1.33 ± 0.21	0.45 ± 0.02	0.32 ± 0.04
G_4_	+64	0.64 ± 0.04	0.38 ± 0.02	0.27 ± 0.02
G_5_	+256	0.22 ± 0.04	0.53 ± 0.09	0.39 ± 0.06

**Table A2:** Effective free energy of binding (∆G_bind,eff_), number of effective protamine /dendrimer positive charges (N_eff_), and effective-charge-normalized free energy of binding (∆G_bind,eff_/N_eff_) for protamine and the different generation EDA-core PAMAM dendrimers (G_1_-G_6_) in complex with heparin as derived from atomistic MD simulations. Adapted from [33] with the permission of The Royal Society of Chemistry.

Heparin Binder	N_eff_	∆G_bind,eff_ (kcal/mol)	∆G_bind,eff_/N_eff_ (kcal/mol)
Protamine	12 ± 1	−3.96 ± 0.41	−0.33 ± 0.04
G_1_	6 ± 1	−1.14 ± 0.22	−0.19 ± 0.05
G_2_	13 ± 1	−16.9 ± 0.5	−1.30 ± 0.11
G_3_	15 ± 1	−15.9 ± 0.3	−1.06 ± 0.07
G_4_	16 ± 3	−14.6 ± 0.8	−0.91 ± 0.18
G_5_	45 ± 5	−18.0 ± 1.3	−0.40 ± 0.05
